# Symptom tracking in endometriosis using digital technologies: Knowns, unknowns, and future prospects

**DOI:** 10.1016/j.xcrm.2023.101192

**Published:** 2023-09-19

**Authors:** Katherine Edgley, Andrew W. Horne, Philippa T.K. Saunders, Athanasios Tsanas

**Affiliations:** 1 EXPPECT and MRC Centre for Reproductive Health, University of Edinburgh, Edinburgh EH16 4UU, Scotland, UK; 2 Centre for Inflammation Research, University of Edinburgh, Edinburgh EH16 4UU, Scotland, UK; 3 Usher Institute, Edinburgh Medical School, University of Edinburgh, Edinburgh EH16 4UX, Scotland, UK; 4 Alan Turing Institute, London NW1 2DB, UK

**Keywords:** endometriosis, digital technology, symptom tracking, wearable technology, chronic pain, smartphone app, smartwatch, patient empowerment

## Abstract

Endometriosis is a common chronic pain condition with no known cure and limited treatment options. Digital technologies, ranging from smartphone apps to wearable sensors, have shown potential toward facilitating chronic pain assessment and management; however, to date, many of these tools have not been specifically deployed or evaluated in patients with endometriosis-associated pain. Informed by previous studies in related chronic pain conditions, we discuss how digital technologies may be used in endometriosis to facilitate objective, continuous, and holistic symptom tracking. We postulate that these pervasive and increasingly affordable technologies present promising opportunities toward developing decision-support tools assisting healthcare professionals and empowering patients with endometriosis to make better-informed choices about symptom management.

## Graphical abstract

**Figure:**
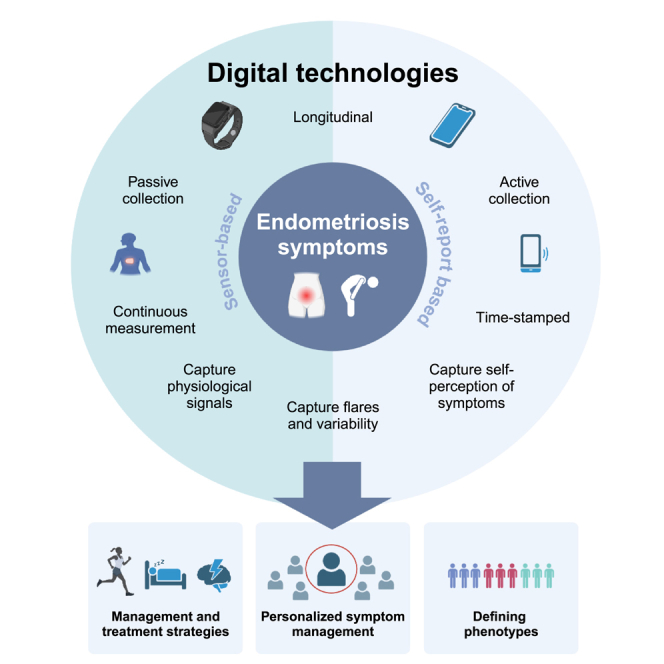

## Teaser

Edgley et al. discuss the potential of digital technologies to facilitate personalized endometriosis symptom tracking and management, informed by applications in similar chronic pain conditions. Research into endometriosis would particularly benefit from data collected remotely to assess longitudinal day-to-day symptom variability, understand heterogeneous symptom trajectories, and inform management strategies.

## Introduction

Endometriosis is a condition that affects an estimated 10% of women of reproductive age and is associated with a range of debilitating symptoms including chronic pelvic pain, fatigue, and infertility.^1^^,^^2^ Endometriosis has a profound impact on quality of life (QoL), with potentially detrimental effects on many aspects of daily living, including work, social life, sexual relationships, self-esteem, and psychological well-being.^3^ Symptom management is often practically challenging, particularly when awaiting a diagnosis, which takes 7–9 years on average.^4^ Standard treatments involve surgery or medical management with hormone-suppressing drugs that may also impact QoL.^5^ Unfortunately, the recurrence rate of symptoms such as pelvic pain after surgical treatment is high (an estimated 40%–50% after 5 years), and many individuals have repeated surgeries.^1^^,^^5^ Thus, identifying effective non-surgical and non-medical strategies for managing the symptoms of endometriosis and their wider impacts on well-being remains a top priority for research into the condition.^6^ In this perspective, we consider the opportunity offered by new, wearable, and other non-invasive monitoring systems to complement and extend the information from traditional patient-reported outcome measures (PROMs) in endometriosis by summarizing some of the evidence of their effectiveness in symptom management from a range of related chronic pain disorders.

## An evolution of methods for symptom tracking

**Figure 1:**
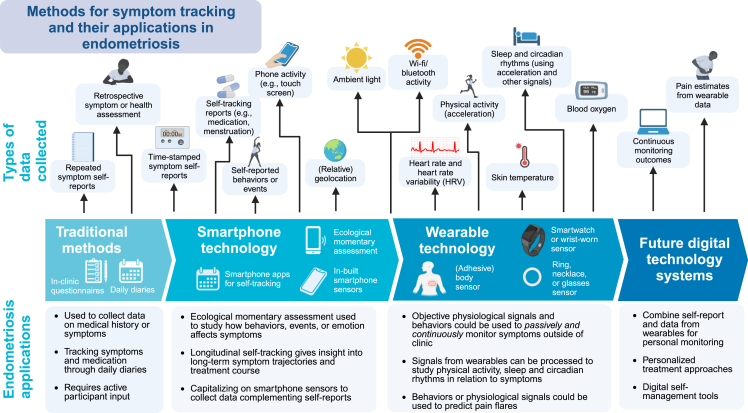
Methods for symptom tracking and their potential applications in individuals with endometriosis symptoms Adapted from “The Drug Discovery Process” by Eunice Huang using BioRender.com (2023). Retrieved from https://app.biorender.com/biorender-templates.

Advances in wearable and smartphone-based technologies have made longitudinal tracking of symptoms and other health measures both accessible and acceptable, as they are generally easy to incorporate into everyday life, allowing collection of data that provide insights into physical and mental health over days and months (Figure 1).^7^ To date, these technologies have not been widely applied in the evaluation of endometriosis symptoms compared with other chronic conditions.^8^^,^^9^ Since symptoms, including unpredictable pain flares, reported by patients with endometriosis have clear parallels with other chronic pain conditions such as irritable bowel syndrome (IBS), migraine, and fibromyalgia,^1^^,^^10^ we can gain useful insights by considering what lessons have been learned when applying digital technologies in those conditions. In the following sections, we review the current traditional methods as applied to endometriosis research, smartphone and wearable technologies, and how these have evolved. We believe that learning from best practice and from the evidence of what has worked well in informing care for related chronic pain conditions can directly inform the development of custom-based apps and tailor the use of similar technologies for endometriosis research.

### Traditional approaches to self-reporting

In endometriosis, where symptoms such as pain and fatigue cannot be objectively assessed, self-reported measures (PROMs) may be used to evaluate the efficacy of treatments or interventions.^11^ Typically, these symptoms are assessed in clinic using standardized questionnaires. However, these traditional methods of self-reporting (see Figure 1) have practical limitations: they require patients to report on symptoms retrospectively, introducing recall bias, and reporting may be influenced by factors such as mood or symptom severity at the time of reporting.^12^ In addition, when self-reporting on sleep, physical activity, or other behaviors that can be captured objectively, retrospective reports may not accurately reflect objective reports.^13^^,^^14^ Alternative ecological momentary assessment (EMA; see Box 1 for definition) methods such as daily diaries, where the assessment takes place on multiple days in a patient’s normal environment, can minimize the influence of recall bias in many cases.^15^^,^^16^ However, if the diaries are completed on paper, it is often difficult to ensure that self-reports are completed at the designated time, and manually digitizing the data prior to analysis is burdensome.^12^

Box 1 **Ecological momentary assessment (EMA)**

**Figure 2:**
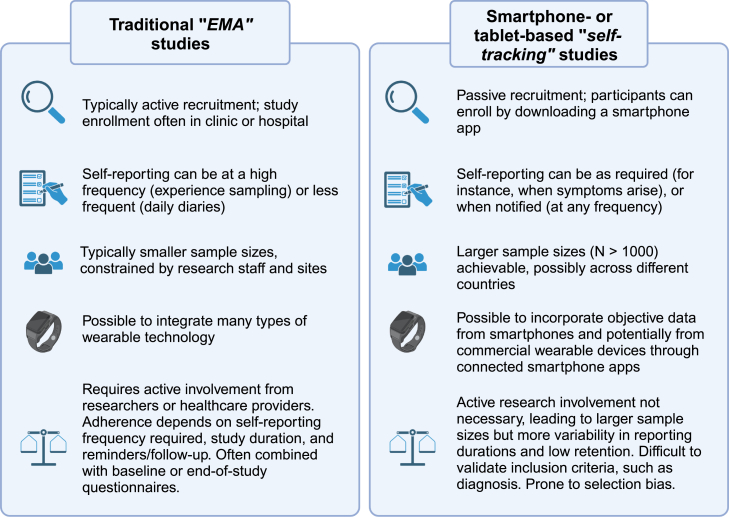
A comparison of self-reported symptom-tracking methods Traditional (non-virtual) studies collecting ecological momentary assessment (EMA) data are described on the left-hand side; virtual or smartphone- or tablet-based studies (termed “self-tracking” here) are described on the right-hand side. Created with BioRender.com.

EMA is a method of assessment where data are collected repeatedly from participants in their normal environment in real time (or close to real time).^12^ EMA encompasses methods such as daily diaries, where self-reports for each day are completed, and experience sampling, where emotions or experiences are self-reported (often multiple times a day). EMA may also include repeated physiological measurements, for instance using wearable technology (also referred to as ambulatory monitoring), or the tracking of behaviors (self-tracking). For easier illustration and categorization, here we differentiate traditional EMA methods—daily diaries and experience sampling—from self-tracking methods (Figure 2) and the use of wearable technologies (Figure 1). All methods are considered under the umbrella term symptom tracking.

### Digital approaches to self-reporting

There has been an increasing use of smartphone- or web-based technologies to complement or replace traditional questionnaire evaluations, as these can minimize bias and burden on participants and healthcare providers (Figure 1). Notably, by making questionnaires available online (through website platforms or smartphone-based apps), patients can complete reports in their own time without being constrained to clinic attendance. Furthermore, the development of custom-built smartphone apps has also allowed for improved methods for conducting EMA studies, providing timestamped, real-time data directly to researchers. In some EMA studies, often termed “experience sampling” studies, repeated questionnaires are completed multiple times per day, over several days or weeks, to examine temporal effects.^12^ These studies typically require handheld devices that can prompt participants to enter data, which is easily enabled through smartphone technology. For instance, experience sampling studies in migraine and IBS have utilized smartphones to study the role of emotion or stress in relation to symptoms.^17^^,^^18^^,^^19^

Additionally, electronic or smartphone app-based daily diaries can be useful for examining the course of symptoms or behaviors over time and have been used in migraine and IBS to analyze triggers^20^^,^^21^ and evaluate treatment effects.^22^^,^^23^ Alternatively, symptoms or behaviors can be reported over considerably longer periods through smartphone apps made publicly available through app stores (Figure 2), which can potentially be combined with the tracking of medications, electronic health records, hospital admissions, or other pertinent information. Self-tracking, or “symptom journal,” smartphone apps also have the potential to engage much larger populations through in-app consent processes. These apps offer greater flexibility compared with traditional methods, prompting participants to track different symptoms or behaviors as required. In migraine, commercial self-tracking apps have facilitated large studies into potential triggers,^24^ the impact of symptoms on QoL,^25^ and evaluating interventions.^26^

### From subjective to objective symptom tracking

Employing wearable devices that can passively collect objective, longitudinal data on a diverse range of physiological signals and behaviors also has the potential to complement self-reported methods. Notably, many of the signals collected from wearable technology (see Figure 1) can be processed to extract features that may be clinically informative. For example, these features may provide clinically actionable insights into the activities of daily living (ADLs) or behaviors such as physical activity or sleep. Using actigraphy, or three-dimensional acceleration (accelerometry) data collected from a wearable device, we can objectively infer physical activity and sleep patterns toward obtaining detailed 24-h profile assessments.^27^^,^^28^^,^^29^

Additionally, smartphones may also act as a type of wearable device by providing built-in sensors or modalities that potentially offer further insight into behaviors and symptoms. For instance, many smartphones contain sensors that detect ambient light as well as movement, which can be used to evaluate physical activity,^30^ and device activity in combination with movement or light may provide an indication of behavioral states such as sleep.^31^ Indicatively, one smartphone-based self-tracking study of individuals with chronic pain (grouped into participants with and without fibromyalgia) utilized step counts recorded by smartphones in combination with questionnaires to analyze the relationship between physical activity and pain.^32^ While Bluetooth or relative geolocation could potentially aid in detecting social behaviors or mood,^7^^,^^33^ these modalities may be limited by how smartphones are used or carried (e.g., if participants do not continuously carry them) and hence serve to motivate further the use of wearable devices.

Reports from several studies show that wearable devices provide not only new data modalities but also more accurate, unbiased measurements than self-reported measures of sleep and physical activity.^13^ For example, studies comparing accelerometer-based estimates of physical activity with a retrospective physical activity questionnaire in patients with fibromyalgia reported low to moderate agreement between the two methods.^34^^,^^35^ Similarly, discrepancies between actigraphy-assessed and self-reported sleep have been observed in patients with fibromyalgia, which may be influenced by factors such as fatigue, sleep quality, age, or opioid use.^14^^,^^36^^,^^37^

Using wearable devices collecting actigraphy data, studies have also been able to identify objective sleep differences in patient populations. For example, greater levels of sleep disturbance as measured by actigraphy have been reported in patients with IBS and fibromyalgia when compared with healthy controls.^38^^,^^39^^,^^40^ These results demonstrate that using wearable devices to quantify sleep and physical activity behaviors may support objectively characterizing differences across patient groups, avoiding bias resulting from PROMs, and providing insight into factors that may contribute to this bias. Additionally, passive collection of data using wearable technology that eliminates the need for cumbersome regular self-reporting may facilitate collecting information that can lead to improved assessment of the outcome of longer-term interventions.

### Monitoring and predicting symptoms through wearable technology

In addition to identifying differences between healthy and chronic pain populations, objective measures extracted from wearable or digital devices can be assessed alongside self-reported measures of symptoms or QoL, providing insight into symptom trajectories and the relationship between objective behaviors and subjective symptoms. One large study (N = 419) of women with fibromyalgia found that less sedentary time and more light physical activity was associated with less severe symptoms, including pain and fatigue, though associations with pain varied depending on which questionnaire or measurement was used.^41^ A further study of women with fibromyalgia did not find similar associations between physical activity and pain but rather only with movement-related symptoms (physical QoL, physical function, and movement fatigue).^35^ Since these studies did not examine temporal effects, relying on retrospective PROMs and pain pressure thresholds to evaluate symptoms, further work is needed in this area to assess longitudinal trajectories.

Actigraphy has also been used to study sleep and diurnal rhythms in fibromyalgia. While one study found that averaged objective and subjective sleep measures were not associated with pain symptoms in fibromyalgia,^42^ a larger study of 292 patients with fibromyalgia found significant relationships between activity rhythms assessed using actigraphy and symptom outcomes.^43^ In the larger study, more delayed activity rhythms and lower variability in movement were associated with more severe symptoms, suggesting the importance of investigating not only overall sleep behaviors but also sleep patterns and diurnal rhythms, which are often not considered or recorded in conventional studies.

In IBS, sleep disturbance measured using actigraphy was associated with more severe abdominal pain symptoms.^39^ In migraine, actigraphy has also been used to study temporal associations between sleep and migraine attacks; analyses of a dataset of 98 adults with episodic migraine found a slight increase in objective sleep duration, but not sleep disturbance, following a migraine.^44^ Further analyses found that lower sleep quality increased the odds of a migraine the following day, although the association was present only for subjective, but not objective, sleep quality.^45^ Additionally, a study of women with chronic migraine used actigraphy along with circadian phase assessment to examine circadian rhythms; the study found that circadian misalignment may worsen migraines, irrespective of sleep duration.^46^

Furthermore, a small study of women undergoing hysterectomy for benign gynecologic conditions used actigraphy data recorded before and after surgery to investigate the impact of sleep on recovery; their findings suggest that better sleep before surgery was associated with lower post-operative pain.^47^ Wearable technology has also been used to monitor sleep changes resulting from lifestyle interventions in fibromyalgia,^48^ demonstrating that wearable sensors can provide useful outcome measures for assessing interventions.

## Use of digital technologies in research on endometriosis

As highlighted above, in studies on fibromyalgia, IBS, and migraine, a range of digital technologies have been used to track symptoms and have provided insights that may help in the management or understanding of these chronic pain conditions. While a number of well-validated questionnaires have been used to gather valuable PROMs in patients with, or awaiting a diagnosis of, endometriosis, they may also be cumbersome for participants and less amenable to application for longitudinal studies.^11^^,^^49^ Wearable devices, in contrast, offer an opportunity to collect granular, objective data for longer periods of time and could be used in conjunction with PROMs to provide new insights into symptoms and response to therapies in endometriosis. Despite these apparent advantages, few studies have explored these technologies in patients with endometriosis, and to our knowledge, no studies have utilized wearable technology to objectively report on endometriosis symptom trajectories or interventions.

However, digital technologies have previously been used in endometriosis for studies that require the monitoring of symptoms to evaluate the efficacy of medication or therapies.^11^ For instance, clinical trials in endometriosis have utilized daily electronic pain diaries to evaluate hormonal therapies such as gonadotropin-releasing hormone (GnRH) antagonists,^50^ and several electronic daily PROMs have been developed for the assessment of interventions in endometriosis.^51^^,^^52^ Daily symptom reports allow for hormonal treatments, where the effect may not be immediate or change over time, to be assessed longitudinally in endometriosis. Electronic versions of these symptom reports may be more suitable for clinical trials with long study durations, as participants can be notified to timely track symptoms, and retrospective reporting can be prevented or recorded.^53^ In addition, EMA administered through smartphones has also been used for other purposes in endometriosis: one study utilized a smartphone app to repeatedly record abdominal pain symptoms and emotional state in patients with endometriosis (N = 34) and unmatched healthy controls (N = 31), reporting statistically significant associations between mood and pain symptoms that were weaker in the control group.^54^ That study demonstrates the importance of exploring within-person effects that cannot be adequately captured through cross-sectional studies and that could be invaluable in providing information to inform improved symptom management strategies.

One large-cohort study involved over 4,000 participants with endometriosis self-tracking their symptoms and treatment through the “Phendo” app for over 2 years.^55^ This free-to-use smartphone app is part of Citizen Endo, a citizen science initiative that is claimed to have over 15,000 users (https://citizenendo.org/research/). Through the Phendo app, participants can track pain symptoms (by providing location, description, and severity), gastrointestinal (GI) or urine issues, and a broad range of other symptoms including those related to common comorbidities of endometriosis.^9^^,^^55^ In the published cohort study, the researchers extracted phenotypes based on the symptoms, medication usage, mood, and other characteristics from the participant data, which they reported were well aligned to expert classifications for the mild and severe phenotypes when examining cluster purity.^55^

Other analyses of data from the Phendo app have explored prevalence and variability of symptoms: from a sample of over 6,000 Phendo participants, they found a high prevalence of both pelvic pain and GI problems.^9^ In addition, that study found high within-person variance in daily pain, indicating that pain varied not only between participants but also varied substantially over time for individual participants, suggesting the relevance of tracking endometriosis-associated symptoms longitudinally through digital technologies. A further study capitalizing on the data collected from the Phendo app (N = 1,009) examined associations between self-reported exercise and pain symptoms, where participants were able to report exercise daily (yes/no) and input the type of exercise as free text.^56^ The authors used generalized linear mixed models to predict daily pain scores and change in pain scores, finding that habitual exercise may moderate the relationship between exercise and next-day pain. A further associated study (N = 52) found that the daily self-reports of exercise (yes/no) through the Phendo app were moderately correlated with self-reported step counts and minutes in moderate-to-vigorous intensity exercise (MVE) (derived from body-worn accelerometers).^57^ However, the limitations of self-reporting exercise without quantification of intensity supports the use of incorporating wearable technology to objectively track exercise, as it can be recorded passively without the need for self-reporting.^58^

In addition to the Phendo app, ongoing studies as part of the Citizen Endo research project also include Phendo Voice, which involves daily voice recordings from participants over 6 weeks to give insight into whether voice characteristics may be indicative of symptoms in endometriosis and whether they can potentially be used to predict pain flares (https://citizenendo.org/research/). Other applications of digital technologies for endometriosis include self-tracking apps such as the Lucy app (https://hellolucy.app/en), developed to track menstruation and symptoms to ultimately provide guidance on possible diagnoses, including endometriosis. One self-tracking app for recording cannabis use has also been used to explore intervention effects in a subset of participants with endometriosis.^59^ Lastly, a small pilot study of patients with endometriosis (N = 3) explored sleep changes before and after laparoscopic surgery using a non-contact sensor installed at home,^60^ which may be an alternative way of collecting data from patients and of overcoming barriers with self-reports or the use of digital technologies that might be challenging for some participants to use (e.g., post-operatively).

## Bringing digital technologies into endometriosis research: Considerations and limitations

While digital collection of PROMs as well as wearable technologies can serve as important tools in studying chronic pain conditions such as endometriosis, to leverage these technologies effectively so that they can provide valid insights and management approaches, studies need to consider and to mitigate against the limitations of these tools. As with other patient-centered activities, research using digital technologies needs to follow best practice in the processing and analysis of symptom-tracking data so that there is not only the opportunity for comparability between studies but also to maximize the utility of these rich data sources for further analysis. Limitations and their mitigation are discussed in the following sections.

### Patient burden and missing data

Symptom tracking through smartphone apps can involve substantial input from participants and therefore, depending on the study procedures, may involve large variability in reporting adherence and larger amounts of missing data than conventional studies.^12^ Reporting patterns may be influenced by various factors; in endometriosis, the severity of pain or fatigue symptoms could feasibly impact reporting patterns of participants, for instance, and thus missing data may introduce bias into symptom-tracking studies that should be investigated to identify any patterns and, ideally, taken into consideration in the analysis. For instance, a study of patterns of missing data among users of a daily diary for endometriosis symptoms found lower completion on certain days of the week.^53^ In long-term smartphone self-tracking studies, reporting could vary from a few days to even years, and therefore levels of certainty in findings will vary. Models that account for this variability, such as those used in Urteaga et al.,^55^ are therefore particularly useful in extracting robust insights from symptom-tracking data. Additionally, an analysis of retention rates in several self-tracking studies across conditions, including the Phendo app, have identified approaches that may help mitigate against low retention, such as clinical referrals and monetary incentives.^61^ Passive systems for symptom tracking through wearable devices thus may be particularly useful in endometriosis to minimize the burden of self-reporting and also bias introduced by missing data.

### Incorporating wearable sensor data into studies

Future study protocols in endometriosis could incorporate wrist- or body-worn sensors into longitudinal studies or clinical trials to objectively assess physical activity, sleep, diurnal rhythms, and other physiological signals in combination with other self-reported PROMs, to provide greater insight into the impacts of interventions and associations between objective measures and symptoms. However, not all wearable devices offer access to the raw data and may instead only provide outputs extracted through proprietary algorithms.^28^ In actigraphy studies, expert assessments by humans, rather than algorithms, may be used to classify sleep and wake periods, though this approach requires expertise and is time consuming and costly.^13^ Ideally, future studies should develop transparent and open-source methods for processing the data from wearables to allow for comparability between studies.^13^^,^^28^

Data from wearable devices often may provide insight not only into overall physical activity, sleep, or measures that can be summarized across days or individuals but also into activity or diurnal rhythms when data are captured over multiple days. Insight into sleep-wake patterns can be obtained through descriptive statistics or non-parametric measures of variability, for instance, or through parametric methods that capture diurnal rhythms, as shown in a previous study of fibromyalgia.^43^ As demonstrated by that study, information on diurnal rhythms that can be extracted from longitudinal wearable data may reveal new insights (over and above sleep duration or sleeping times) in relation to symptoms.

Nevertheless, although actigraphy-assessed sleep-wake or activity rhythms, a measure of diurnal rhythms, may provide insight into intrinsic circadian rhythms (endogenous processes with near 24-h oscillation), they do not necessarily align with circadian rhythms completely.^13^ Further measures may therefore be necessary to assess circadian rhythms, such as circadian phase assessment, as used in a study of migraine,^46^ or body temperature.^62^ Wearable devices that collect heart rate or heart rate variability (HRV) data or body temperature can improve the assessment of circadian rhythms,^63^ although the adoption of these tools for chronic pain conditions is currently limited. Given that there is some indication of a potential link between circadian disruption (for instance, through shift work^64^) and endometriosis that has not been extensively explored, as well as a potential association with symptoms in related conditions such as migraine,^46^ future studies could assess circadian rhythms through new wearable sensor modalities to better explore associations with pain or other symptoms in endometriosis or, ideally, to assess the impact of interventions on sleep, activity rhythms, and circadian rhythms simultaneously.

Integrating data from wearable sensors via smartphone apps with other patient-specific data (e.g., from PROMs and clinical tests) may also be feasible, as demonstrated by a previous study of chronic pain,^32^ and can enable large-scale studies and can potentially be combined with smartphone-based self-tracking (Figure 2). Data from commercial wearable devices may be linked through smartphone apps with the advent of platforms such as ResearchKit or through capitalizing on application programming interfaces (APIs) to collate data. It is important to note that the data collected may exhibit some differences across commercial devices, which could be due to inherent hardware or software settings (an indicative example is using different sample rates when collecting acceleration data). In addition, many smartphones contain built-in sensors to collect information such as step counts; however, we stress that data may not be identical across different smartphones (for example, the built-in hardware might have different sensitivity or resolution). Furthermore, the transparency of the algorithms used may be limited when relying on commercial wearable devices, and other limitations include patient privacy and selection bias (we refer to Dorsey et al. for further commentary on these limitations).^65^ Nevertheless, despite possible inherent differences between sensors (embedded in wearable devices and smartphones), it is often possible to develop algorithmic approaches when processing the data to take these into account and adjust outputs accordingly where required (through testing of devices under similar recording conditions). Therefore, we believe that this approach could be particularly useful in endometriosis for exploring the relevance of physical activity to symptoms in larger populations through community studies where participants would be using their own devices.

Additionally, while data from wearable devices can potentially provide important objective outcome measures in clinical trials for endometriosis, it may be challenging to identify *a priori* which techniques and outcomes would provide clinically meaningful insights when extracting information from wearable devices. Thus, more exploratory studies may be needed before successful incorporation into clinical trial protocols, particularly in endometriosis, where there exists limited objective information on sleep, activity, diurnal rhythms, or other physiological signals and their relationship with endometriosis symptoms.

### Overlapping conditions

Endometriosis is associated with a higher risk of various conditions, including those discussed here—IBS, migraine, and fibromyalgia—in addition to painful bladder syndrome (interstitial cystitis) and certain autoimmune conditions.^1^ Therefore, it can be challenging to differentiate symptoms deriving from overlapping conditions or comorbidities. Ideally, when self-tracking through smartphones, participants could track a broad range of symptoms that may be prevalent both to endometriosis and other conditions and also comorbidities. As it may be difficult for participants to distinguish symptoms from overlapping conditions, this could entail tracking pain by body location as well as GI or urinary systems, an approach used by the Phendo app.^55^ Furthermore, combining flexible self-tracking methods with wearable technology could provide greater insight into different types of endometriosis symptoms (including potentially defining endometriosis subtypes if we can collect large datasets) and the impact of sleep or physical activity, while EMA methods could be useful in understanding interactions between symptoms.

### Analysis methodology

Symptom-tracking data can be challenging to analyze due to its longitudinal, possibly multimodal nature. Multiple data points are collected for each participant spaced out in time and collected at regular or irregular time points, often referred to as repeated-measures or time-series data. Since repeated measures from an individual may be highly correlated, common statistical approaches (for instance, Pearson correlation or linear regression) may be unsuitable, as they often rely on assumptions that can be violated by the fact that the samples are not independent.^66^ Although some statistical methods may be applicable to repeated-measures data collected in interventional studies, alternative methods are often more appropriate for gaining insight into symptom-tracking data collected repeatedly over time. Thus, clinical trials as well as exploratory studies collecting symptom-tracking data typically utilize statistical analysis approaches that account for the correlations present in repeated-measures data, and we refer readers to previous guidance on these approaches.^67^ We provide a very brief overview of commonly used methods below.

A simple approach that may be used to analyze repeated-measures data is to summarize the data from each participant in the dataset, often using descriptive statistics such as mean and standard deviation, to then analyze the resulting data using statistical approaches for independent samples.^66^ This approach provides insight into between-person relationships in the data but overlooks valuable information present within individuals. Alternative approaches, where all repeated-measures data are utilized, include statistical tests and correlation adapted for repeated-measures data or mixed-effects models (also known as multilevel models).^68^

Statistical or machine-learning approaches may be preferable when prioritizing predictive accuracy and typically utilize separate test data or K-fold cross-validation, and potentially an external validation dataset, to evaluate how well models perform on new, unseen data. Machine-learning models may be more suitable for complex and high-dimensional data but in certain cases lack interpretability, which may be essential for applications that aim to provide insight into diagnosis, management, or impacts of a condition such as endometriosis. That said, there also exist approaches to improve interpretability: for instance, feature selection methods aim to identify the jointly most predictive feature set among all possible features, and this is a very active area of ongoing research.^69^^,^^70^ In addition, certain machine-learning models have integrally embedded the computation of feature importance scores (e.g., random forests), and other analysis tools such as SHAP values can help identify important predictors.^71^ Machine-learning approaches may be particularly useful for developing clinical support tools or personalized prediction models, and we refer to current guidelines on the development and reporting of these tools when implemented for clinical practice.^72^^,^^73^

Lastly, other analyses of symptom-tracking data include the clustering of trajectories to gain insight into subgroups or phenotypes within the data, as explored in both endometriosis and fibromyalgia.^55^^,^^74^ With a sufficiently large number of participants, symptom-tracking data may be particularly useful to reveal new clusters of participants with potential clinical relevance, i.e., defining patient phenotypes which might lead to more targeted monitoring of individuals who are given an umbrella diagnosis (it is known there are different endometriosis subtypes based on the location of lesions,^1^ but there may be further ways of categorizing patients based on symptom presentation, for instance^55^). Methods such as k-means or hierarchical clustering can be applied to summarized time series from each participant, or, alternatively, these can be combined with methods (distance measures) that utilize the entire time series for each participant.

## Future directions in endometriosis research and translation for patient benefit

Digital technologies offer new approaches for monitoring and studying chronic conditions with diverse symptomology, such as endometriosis, that are characterized by heterogeneous symptom trajectories and different optimal treatment strategies. For patients with chronic pain that may be associated with endometriosis, long delays in receiving a diagnosis and lack of effective pain treatment have increased the interest in developing approaches for self-management, which include non-surgical interventions such as lifestyle changes.^6^ As demonstrated by several studies of patients with symptoms associated with fibromyalgia, migraine, and IBS, wearable and smartphone technology can be used to monitor both pain symptoms (including pain flares) alongside other behaviors, including sleep, physical activity, and diurnal rhythms, or contextual factors such as emotional state or stress. The findings generated from these data can not only provide insight into potential management strategies but also identify patterns among heterogeneous symptom trajectories that could ultimately help provide more individualized care based on a person’s “digital” phenotype.

Currently, more research around using these tools in the endometriosis patient population is urgently required before we can use these technologies toward informing patient healthcare. Specifically, using machine-learning methods to predict PROMs using data from wearables may enable improved prediction of changes in symptom severity or onset of flares to help not only in self-management strategies but also in unbiased and more rapid evaluation of interventions. Additionally, data from wearable devices could be used to impute missing data in PROMs as well as identify or minimize bias from PROMs. New wearable technology solutions also have the potential to passively assess pain intensity,^75^ thus allowing for improved comparison and monitoring of the pain levels patients experience, which can be difficult on subjective scales that are influenced by recall and self-reporting bias. In particular, clinical trials in endometriosis commonly use PROMs to assess pain levels, in which it can be difficult to compare subjective pain ratings between participants (a pain score of “4” for one person may be similar to a “6” for another) and even between reports by the same participant. Therefore, employing wearable technologies together with subjective outcome measures could improve the quality of assessment in clinical trials, for instance through creating “personalized pain scales”^75^ or by providing additional objective outcome measures, thus limiting bias and missing data from subjective PROMs and minimizing the burden on participants. Combining both wearable and smartphone technology for tracking symptoms longitudinally would also provide a rich source of data for understanding heterogeneous symptom trajectories, and, ultimately, multimodal data (including from digital technologies, e.g., to complement traditional clinical tests) could inform the development of self-management strategies and help optimize treatments.

Furthermore, while the use of digital technologies to manage and understand endometriosis has begun to advance, the applicability of symptom tracking to the diagnosis of endometriosis has not been extensively explored or validated. Researchers have previously designed endometriosis screening tools that rely on patient-generated questionnaire data, often combined with medical history or other clinical data.^76^^,^^77^ However, few approaches have incorporated longitudinal self-tracking of symptoms, though it is likely that questionnaire-based screening tools could be improved by utilizing real-world information from patients on their longitudinal symptom trajectories, the effect of medications, and QoL, which could be enabled through smartphone-based symptom tracking. Wearable technology could potentially enhance this rich data source by providing further longitudinal symptom or physiological variables, although the utility of wearable-derived information toward diagnosis or screening endometriosis is currently unknown. Collectively, these multimodal, longitudinal data, potentially along with normative data and related data patterns from patients with confirmed clinical diagnosis of endometriosis, could be conveniently presented to expert clinicians. Ultimately, however, we want to emphasize that these digital health tools are not there to replace clinicians: we view their potential as empowering clinicians facilitating informed decision-making.

### Conclusions

Symptom tracking through wearable and smartphone technology can provide insights into disease symptom trajectories, which can be complementary to elicited PROMs by capturing data longitudinally and at a high granularity. In chronic conditions that lead to a broad symptom constellation, similar to endometriosis, digital technologies have been used to characterize physical activity and sleep behaviors, identify potential symptom triggers, work toward defining subtypes of the condition, and assess interventions. However, limitations of self-tracking through PROMs, such as diversity in reporting duration and analysis challenges, need to be carefully considered for future applications in endometriosis. Although recent studies of endometriosis employing self-tracking using smartphones have shown promise toward understanding heterogeneous symptom trajectories and symptom self-management, incorporating wearable technology would allow for capturing objective data passively and longitudinally, which could provide new insights into the condition as well as address shortcomings of self-reported data. Employing, and ideally combining, both subjective and objective symptom-tracking data has the potential to help healthcare professionals and patients with endometriosis better predict and manage pain flares, reduce recall bias, optimize and monitor response to treatments, and inform self-management strategies.
